# Optimization of chemical transfection in airway epithelial cell lines

**DOI:** 10.1186/s12896-025-00945-x

**Published:** 2025-01-23

**Authors:** Tony J. F. Guo, Wan Yi Liang, Gurpreet K. Singhera, Jasmine Memar Vaghri, Janice M. Leung, Del R. Dorscheid

**Affiliations:** 1 Centre for Heart Lung Innovation, St. Paul’s Hospital, Providence Healthcare Research Institute, University of British Columbia, 1081 Burrard St, Vancouver, BC V6Z 1Y6 Canada; 2 Department of Medicine, University of British Columbia, 2775 Laurel St, Vancouver, BC V5Z 1M9 Canada

**Keywords:** Airway epithelium, Chemical transfection, Optimization

## Abstract

**Background:**

Chemical transfection is a widely employed technique in airway epithelium research, enabling the study of gene expression changes and effects. Additionally, it has been explored for its potential application in delivering gene therapies. Here, we characterize the transfection efficiency of EX-EGFP-Lv105, an EGFP-expressing plasmid into three cell lines commonly used to model the airway epithelium (1HAEo-, 16HBE14o-, and NCI-H292).

**Results:**

We used six common and/or commercially available reagents with varying chemical compositions: Lipofectamine 3000 (L3000), FuGENE HD, ViaFect, jetOPTIMUS, EndoFectin, and calcium phosphate. Using L3000, 1HAEo- exhibited the highest transfection efficiency compared to 16HBE14o- and NCI-H292 (1HAEo-: 76.1 ± 3.2%, 16HBE14o-: 35.5 ± 1.2%, NCI-H292: 28.9 ± 2.23%). L3000 yielded the greatest transfection efficiency with the lowest impact on cellular viability, normalized to control, with a 11.3 ± 0.16% reduction in 1HAEo-, 16.3 ± 0.08% reduction in 16HBE14o-, and 17.5 ± 0.09% reduction in NCI-H292 at 48-hour post-transfection. However, jetOPTIMUS had a similar transfection efficiency in 1HAEo- (90.7 ± 4.2%, *p* = 0.94), but had significantly reduced cellular viability of 37.4 ± 0.11% (*p* < 0.0001) compared to L3000. In 16HBE14o-, jetOPTIMUS yielded a significantly higher transfection efficiency compared to L3000 (64.6 ± 3.2%, *p <* 0.0001) but significantly reduced viability of 33.4 ± 0.09% (*p <* 0.0001) compared to L3000. In NCI-H292, jetOPTIMUS yielded a lower transfection efficiency (22.6 ± 1.2%) with a significant reduction in viability (28.3 ± 0.9%, *p* < 0.0001). Other reagents varied significantly in their efficiency and impact on cellular viability in other cell lines. Changing the transfection mixture-containing medium at 6-hour post-transfection did not improve transfection efficiency or viability. However, pre-treatment of cell cultures with two rinses of 0.25% trypsin-EDTA improved transfection efficiency in 1HAEo- (85.2 ± 1.1% vs. 71.3 ± 1.0%, *p* = 0.004) and 16HBE14o- (62.6 ± 4.3 vs. 35.5 ± 1.2, *p* = 0.003).

**Conclusions:**

Transfection efficiencies can differ based on airway epithelial cell line, reagents, and optimization techniques used. Consideration and optimization of cell line and transfection conditions may be useful for improving nonviral genetic techniques in vitro.

**Supplementary Information:**

The online version contains supplementary material available at 10.1186/s12896-025-00945-x.

## Background

The airway epithelium functions as a physical and immune barrier between the external environment and interstitial lung tissue to prevent the invasion of pathogens, allergens, and other noxious compounds [1]. In recent years, it has become a focal point for investigation, particularly due to its central role in mediating COVID-19 [2], and chronic respiratory diseases such as asthma [3] and chronic obstructive pulmonary disease [4]. In vitro airway epithelial models are useful to study the pathophysiology of diseases such as asthma, chronic obstructive pulmonary disease, and cystic fibrosis, as well as for testing the efficacy of pharmacological compounds. Manipulating the expression of proteins of interest allows for the investigation of cellular processes and molecular mechanisms. This manipulation can take the form of transfection, a process whereby foreign nucleic acids are introduced into eukaryotic cells to either increase or decrease the expression of a protein [5]. However, the airway epithelium is inherently resistant to invasion by foreign particles, including pathogens and polymer- and lipid-based nanoparticles, due to the mucus and immunological barrier present [6, 7], making these type of studies difficult and variable. Therefore, optimization of transfection in airway epithelial models is essential to ensure generalizability of results.

Expression of the transgene can be stabilized by integrating exogenous DNA into the host genome, allowing long-term expression. A common approach to achieve stable expression is through viral transfection, particularly with lentiviral vectors. These vectors can integrate its genome into both dividing and non-dividing cells and possesses broad cellular tropism [8]. This contrasts with transient expression, whereby DNA delivered into the nucleus is transcribed but not integrated, leading to a reduction in transgene expression over time [9]. A common method for transfection is to use chemical reagents such as cationic lipids and polymers. Cationic lipids such as lipofectin form liposomes that encapsulate nucleic acids to facilitate their entry into cells via endocytosis [10]. Cationic polymers such as polyethyleneimine (PEI) can interact with anionic phosphates of DNA to condense it and form complexes that can be internalized by cells to mediate delivery [11]. Other forms of non-viral gene delivery systems exist, including the use of inorganic nanoparticles, hybrid systems combining different types of non-viral vectors, and physical methods such as electroporation [12, 13]. Chemical transfection in mammalian cells remains widely used; however, it can cause cytotoxicity, making optimization essential to achieve acceptable transfection efficiencies.

In this study, our objective was to assess the transfection efficiency and cellular viability resulting from the use of various commercially available reagents in three commonly used cell lines modeling the airway epithelium. Five commercially available transfection reagents with proprietary formulations were used: Viafect and jetOPTIMUS, both cationic reagents; FuGENE HD, a non-liposomal lipid blend; and EndoFectin and Lipofectamine 3000, both lipid-based reagents [5, 14]. As these are proprietary, the exact composition and mechanism of action is not clear. Calcium phosphate precipitation was also tested. Furthermore, we evaluated various parameters that may affect the transfection efficiencies and cellular viability. This research aims to provide optimization techniques for researchers working with transfection in airway epithelial models, which are often challenging to transfect.

## Methods

### Reagents and plasmid

ViaFect and FuGENE HD (FuGENE) were obtained from Promega (Madison, WI). jetOPTIMUS was obtained from Polyplus (Illkirch, France). Lipofectamine 3000 (L3000) was obtained from Thermo Fisher Scientific (Waltham, MA). EndoFectin Max was obtained from GeneCopoeia (Rockville, MD). Calcium chloride was obtained from Sigma-Aldrich (St. Louis, MO). alamarBlue resazurin-based cell viability solution was purchased from Thermo Fisher Scientific.

EX-EGFP-Lv105, a mammalian expression plasmid expressing enhanced green fluorescent protein (eGFP) was obtained from GeneCopoeia. After transformation into chemically competent NEB Stable cells (New England Biolabs, Ipswich, MA), a single colony was picked and propagated in liquid Luria-Bertani (LB) broth with 10 µg/mL of ampicillin. The plasmid was isolated using the PureYield plasmid Maxiprep kit (Promega) and resuspended in nuclease-free water. Plasmid concentration and purity was determined using a NanoDrop spectrometer (Thermo Fisher Scientific). The plasmid was aliquoted into smaller volumes and frozen at −20 °C until it was ready for use.

### Cell culture

16HBE140- (16HBE) and 1HAE0- (1HAE) airway epithelial cells were obtained as a generous gift from the laboratory of Dr. Dieter Gruenert at the University of California San Francisco. 16HBE cells are immortalized through simian virus 40 (SV40) T antigen transformation and are often used as a wild-type control in the study of cystic fibrosis [15, 16]. 1HAE is immortalized using an SV40 plasmid and retains junctional and ion transport characteristics [17, 18]. NCI-H292 airway epithelial cells were derived from mucoepidermoid carcinoma and obtained from the American Type Culture Collection (Manassas, VA). NCI-H292 have been used to investigate inflammatory responses to cigarette smoke [19, 20]. All cell lines were seeded onto T25 flasks (Cell + T25 flasks, Sarstedt, Germany) using Dulbecco’s Modified Eagle’s Medium (DMEM, #11995065, Thermo Fisher Scientific) supplemented with 10% fetal bovine serum (FBS, A3840302, Thermo Fisher Scientific) and 1% penicillin-streptomycin (#10378016, Thermo Fisher Scientific), which together are referred to as complete DMEM. Cells were sub-cultured at 70% confluence every 2–3 days. Cell stocks were frozen in complete DMEM with 10% dimethyl sulfoxide and stored at −80 °C.

### Transfection

Cells were transfected based on manufacturer’s recommended instructions. At 70–80% confluency, cell cultures on T25 flasks were briefly washed with warm Dulbecco’s phosphate buffered saline (PBS, #14190144, Thermo Fisher Scientific). Trypsin-EDTA, 0.25% (#25200056, Thermo Fisher Scientific) was added to the flask and incubated at 37 °C for 7–10 min. Trypsin was neutralized with an equal volume of complete DMEM. Cells were pelleted at 200× g for 7 min and resuspended in complete DMEM. All transfections were performed on a 48-well plate (Cell + 48 well cell culture plate, Sarstedt) at a seeding confluency of 2.5 × 10^4^ cells per well (1 × 10^4^ cells/mL, or 2.27 × 10^4^ cells/cm^2^). Transfections were done within 18–24 h of seeding when the cultures had an estimated confluency of 40%. 2.5 µg of plasmid DNA was used for transfection per well. For the Opti-MEM incubation treatment, the media within the wells were aspirated and wells were once rinsed with Opti-MEM (#31985062, Thermo Fisher Scientific). Subsequently, Opti-MEM was added and transfection was performed.

Liposomal reagents, such as L3000, Viafect, and EndoFectin, and non-liposomal formulations such as FuGENE and jetOPTIMUS were used [5]. Calcium phosphate (CaP), which involve the precipitation of DNA with calcium ions to be endocytosed by target cells [21], was also used. The reagent: DNA ratio for each transfection reagent tested was previously optimized by the authors based on manufacturer’s recommendations.

For L3000 transfection, 12.5 µL of Opti-MEM was added into two microcentrifuge tubes each. To one, 0.375 µL of L3000 was added, and to the other, plasmid DNA and 0.5 µL of P3000 reagent were added with a reagent: DNA ratio of 1.5:1. Following a 10-minute incubation at room temperature, the DNA/P3000 mixture was added dropwise into the L3000 mixture and gently vortexed. This mixture was incubated for an additional 12 min at room temperature, followed by the addition of 25 µL dropwise into the wells.

For ViaFect, DNA was added into 10 µL of Opti-MEM and gently vortexed. To that, 2.25 µL of ViaFect reagent was added with a reagent: DNA ratio of 3:1. The mixture was gently vortexed and incubated at room temperature for 12 min before 10 µL of the mixture was added into the wells.

For FuGENE, DNA was added into 10 µL of Opti-MEM and gently vortexed. To that, 1.5 µL of reagent was added with a reagent: DNA ratio of 3:1. The mixture was gently vortexed and incubated at room temperature for 10 min before 10 µL of the mixture was added into the wells.

For jetOPTIMUS, DNA was added into 25 µL of supplied buffer and gently vortexed. To that, 0.25 µL of jetOPTIMUS was added into the buffer at a reagent: DNA ratio of 1:1. The mixture was gently vortexed and incubated at room temperature for 10 min before 25 µL of the mixture was added into the wells.

For EndoFectin, 12.5 µL of Opti-MEM was added into two microcentrifuge tubes each. DNA was added to one and 0.75 µL of EndoFectin to the other. Diluted EndoFectin was added into the DNA containing Opti-MEM at a reagent: DNA ratio of 3:1 and incubated at room temperature for 15 min before 10 µL was added into each well.

The protocol for calcium phosphate transfection was adapted from Kinston et al. [22]. Briefly, DNA was added to 1.3 µL of 2 M CaCl_2_ and diluted with water to a final volume of 10.5 µL. An equal volume of 2X concentrated HEPES-buffered saline was added and incubated at room temperature for 20 min before being added to the wells. HEPES-buffered saline contains 280 mM NaCl, 50 mM HEPES, 1.5 mM Na_2_HPO_4_, and pH was adjusted using NaOH to 7.05.

Media of all wells was replaced with complete DMEM at 24-hour post-transfection except for the treatments where the media was changed at 6 h. Transfections were performed twice.

For trypsin-EDTA pre-treatment, the media prior to transfection was aspirated and rinsed with warmed D-PBS. Then, 0.25% trypsin-EDTA was added, and wells were incubated at room temperature for 10–15 s. This was aspirated and a second trypsin rinse was performed. Complete DMEM was added, followed by the addition of transfection mixture, and the experiment continued.

### Fluorescence microscopy

48-hour post-transfection, the wells were imaged using the EVOS M5000 imaging system (Thermo Fisher Scientific) and the number of GFP positive cells were manually counted by a blinded observer. Representative images were also taken for qualitative observation.

### Alamar blue viability assay

Alamar Blue reagent was dissolved in deionized water and sterile filtered before being diluted to 10% final volume in complete DMEM. The media in the transfected wells were removed and 200 µL of 10% Alamar Blue was added to each well. The cells were returned to a humidified 5% CO_2_ environment at 37 °C for 2 h. Negative control sample was created by adding 100% DMSO to the two respective wells 10 min prior to the addition of Alamar Blue. Following incubation, the media was transferred into a 96-well microplate and read under a SpectraMax i3 spectrophotometer (Molecular Devices, San Jose, CA) with 560 nm excitation and 590 nm emission. Mean fluorescence values are reported.

### Western blotting

At 48-hour post-transfection, the cultures were lysed with lysis buffer (Cell Signaling Technologies, Danvers, MA) supplemented with phosphatase inhibitor, PhosSTOP (Roche, Basel, Switzerland), and protease inhibitor, cOmplete Protease Inhibitior Cocktail (Roche). Lysates were centrifuged to be pelleted. Protein concentration was quantified using the Pierce bicinchoninic acid protein assay (#23225, Thermo Fisher Scientific) as per manufacturer’s instructions. 15 µg of protein was loaded with β-mercaptoethanol containing sample buffer then boiled before loading on a 10% sodium dodecyl sulfate (SDS) gel. Following separation, the proteins were transferred onto a nitrocellulose membrane (Pall Corporation, New York, NY). Membranes were blocked in 5% skim milk diluted in Tris-buffered saline with 0.01% Tween-20 (Sigma-Aldrich) (TBS-T). Following blocking, the membranes were probed overnight with rocking using mouse anti-GFP (MA5-15256, 1:2500, Thermo Fisher Scientific) and β-actin (sc-47778, 1:2000, Santa Cruz, Dallas, TX) as a loading control. The following day, membranes were washed with TBS-T before incubating with secondary anti-mouse horseradish peroxidase (HRP, AS014, 1:2000, Abclonal Technologies, Woburn, MA) at room temperature for 1 h. Following washes with TBS-T, Clarity Western enhanced chemiluminescence substrate (Bio-Rad, Hercules, CA) was used to detect protein on the membrane. Western blots were imaged using the G: BOX imaging system (Syngene, Bangalore, India). To ensure normalization between separate blots, untreated human embryonic kidney 293T cells were lysed and loaded in duplicate on each blot, accounting for differences such as western blot transfer efficiency and exposure time.

### Flow cytometry

At 48-hour post-transfection, the media was aspirated, and the cells were briefly washed with warm PBS. The cells were subsequently dissociated with 0.25% trypsin-EDTA, neutralized with complete DMEM, and centrifuged at 500× g for 7 min. The pellet was resuspended in PBS to a cell density of ~ 25,000 cells/100 µL. The samples were analyzed using the Gallios flow cytometer (Beckman Coulter, Brea, CA) and GFP-expressing cells were gated using control cells following excitation to a 488 nm laser. Data were analyzed using Kaluza Analysis (Beckman Coulter). For each replicate, 15 000 events were run on flow cytometry.

### Data analysis

Western blot densitometry was performed using ImageJ image analysis software (National Institutes of Health, Bethesda, MA). The signal intensity of the protein of interest was normalized to the signal intensity of the loading control, β-actin. Prism 8 (GraphPad, San Diego, CA) was used as the statistical analysis software. To compare multiple treatments to control, one-way analysis of variance (ANOVA) was used with Tukey’s multiple comparisons post-test was performed, unless specified otherwise. For analysis of cellular viability, comparisons were between treatments at 24- and 48-hour post-transfection, without comparing viability across two time-points. Two-way ANOVA with Šídák’s multiple comparisons test was used. The threshold for statistical significance was set at *p* < 0.05.

## Results

**Fig. 1:**
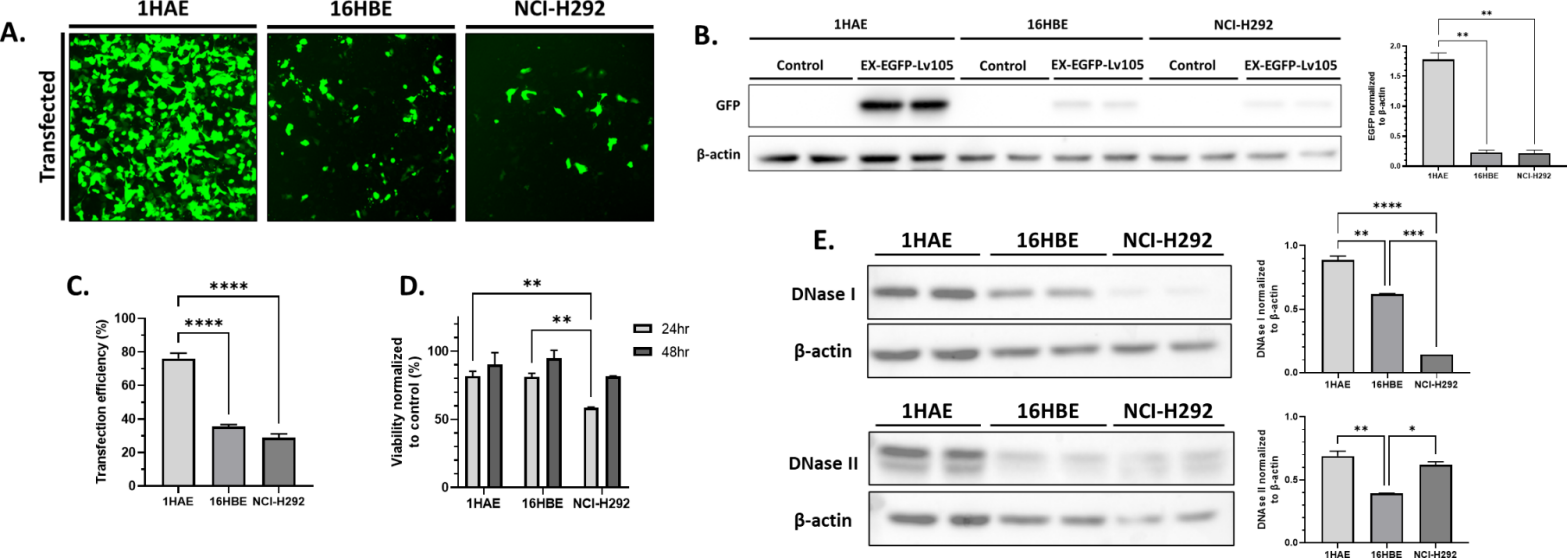
Transfection efficiency 48-hour post-transfection using Lipofectamine 3000 (L3000) varies depending on the airway epithelial cell line used. (**A**) GFP microscopy of transfected cell cultures 48-hour post-transfection. Images are at 100X magnification and representative of transfections done in duplicate, with the experiment conducted twice. (**B**) Western blot of whole cell lysates of 1HAE, 16HBE, and NCI-H292 cultures 48-hour post-transfection probed against GFP (26 kDa) and β-actin (42 kDa) as a loading control. Densitometry analysis is summarized, with GFP expression normalized to β-actin. Only comparisons with *p* > 0.05 are shown. (**C**) Flow cytometry analysis of 48-hour post-transfection. Transfection efficiency represents the percentage of cells gated positive for GFP, compared to control. Data represents two independent transfection experiments. Only comparisons with *p* > 0.05 are shown. (**D**) Alamar Blue cellular viability assay at 24- and 48-hour post-transfection. Fluorescence intensity (560 nm excitation, 590 nm emission) is proportional to the viability of the cell culture. (**E**) Western blot of whole cell lysates of 1HAE, 16HBE, and NCI-H292 was performed and probed against DNase I (31 kDa) and II (40 kDa). Densitometry analysis is summarized, with expression of each DNase normalized to their respective β-actin. For all experiments, ** = *p <* 0.01, **** = *p* < 0.0001. All experiments included two technical replicates and were repeated twice

We characterized the transfection efficiency with Lipofectamine 3000 (L3000), a commonly used transfection reagent, across the three airway epithelial cell lines (Fig. 1). GFP signal as assessed via fluorescence microscopy appeared strongest in 1HAE, followed by 16HBE and NCI-H292 (Fig. 1). Via western blotting and assessment of transfection efficiency using flow cytometry, GFP expression was significantly higher in 1HAE, with no difference between 16HBE and NCI-H292 (Fig. 1B and C). At 24-hour post-transfection, cellular viability was not different between 1HAE and 16HBE but NCI-H292 cellular viability was significantly lower compared to 1HAE and 16HBE. At 48-hour post-transfection, no significant difference in cellular viability was observed across the three cell lines used (Fig. 1D). Deoxyribonuclease I and II (DNase I and II) are an endonuclease and lysosomal enzyme, respectively, that can cleave and degrade DNA which may impact the extent of intact DNA necessary for gene expression [23]. DNase I and II expression levels were evaluated via western blotting in 1HAE, 16HBE, and NCI-H292 cell lines to identify potential differences in expression, which could offer insights into cell line-specific variations in transfection efficiency. DNase I expression was significantly higher in 1HAE compared to both 16HBE and NCI-H292, with 16HBE also showing higher expression than NCI-H292 (Fig. 1E). The same figure shows that DNase II expression did not significantly differ between 1HAE and NCI-H292; however, 16HBE exhibited significantly lower expression than both 1HAE and NCI-H292.

Next, we compared transfection using L3000, FuGENE, ViaFect, jetOPTIMUS, EndoFectin, and CaP in 1HAE, 16HBE, and NCI-H292 airway epithelial cells.

**Fig. 2:**
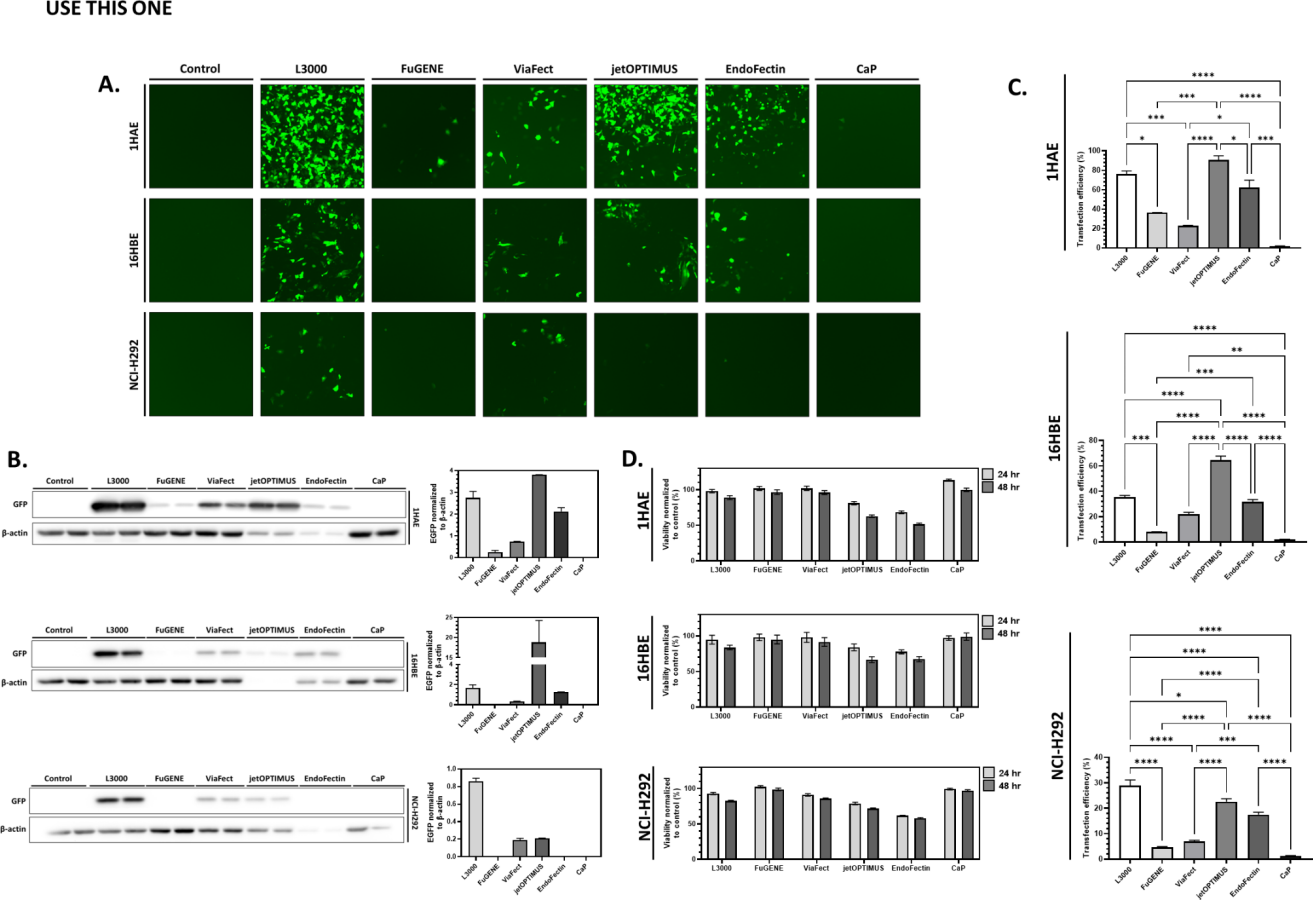
Transgene expression and cellular viability vary between transfection reagents used. (**A**) GFP fluorescence microscopy of 1HAE, 16HBE, and NCI-H292 cell cultures 48-hour post-transfection with Lipofectamine 3000 (L3000), FuGENE HD (FuGENE), ViaFect, jetOPTIMUS, EndoFectin, and calcium phosphate (CaP). Images are at 100X magnification and representative of transfections done in duplicate, with the experiment conducted twice. (**B**) Western blot of whole cell lysates of 1HAE, 16HBE, and NCI-H292 cultures 48-hour post-transfection probed against GFP (26 kDa) and β-actin (42 kDa) as a loading control. Densitometry analysis is summarized, with GFP expression normalized to β-actin. (**C**) Flow cytometry analysis of 1HAE, 16HBE, and NCI-H292 cultures 48-hour post-transfection. Transfection efficiency represents the percentage of cells gated positive for GFP, compared to control. Data represents two independent transfection experiments. Only comparisons with *p* < 0.05 are shown, with pairwise comparisons made between reagents that yielded significantly different transfection efficiencies. (**D**) Alamar Blue cellular viability assay on 1HAE, 16HBE, and NCI-H292 cells 24- and 48-hour post-transfection with the tested reagents normalized to either 24- or 48-hour control. For all experiments, * = *p* < 0.05, ** = *p <* 0.01, *** = *p* < 0.001, **** = *p* < 0.0001. All experiments included two technical replicates and were repeated twice

Using fluorescence microscopy, the transfection efficiency, as assessed by the GFP expression 48-hour post-transfection, was different across reagents and cell lines used (Fig. 2A). Transfection with L3000 and jetOPTIMUS appeared to have the greatest signal in 1HAE, qualitatively through fluorescence microscopy. However, transmitted light microscopy images revealed a significantly higher occurrence of cell death with jetOPTIMUS (Supplementary Fig. 1A). Morphologically, the cells appeared circular and smaller in size, in contrast to the stellate and flattened shape of healthy cells, suggesting they may be unhealthy or dying (Supp. Figure 1B). There was also a greater proportion of dead or dying cells coupled with a decrease in culture confluency with EndoFectin transfection (Supp. Figure 1A). CaP and FuGENE reagents showed low transfection efficiency, with cultures comparable to control based on cell morphology. ViaFect demonstrated intermediate efficiency between L3000 and EndoFectin. In 16HBE, L3000 and jetOPTIMUS exhibited the highest GFP signal but jetOPTIMUS and EndoFectin caused significant cell death and reduced confluency. L3000 and ViaFect provided the greatest GFP signal in NCI-H292, but significant cell death was observed with L3000, ViaFect, jetOPTIMUS and EndoFectin (Supp. Figure 1).

48-hour post-transfection, 1HAE, 16HBE, NCI-H292 cultures were lysed, resolved using SDS-PAGE, transferred, and probed for GFP and β-actin (Fig. 2B). GFP expression is greatest with L3000 transfection in all three cell lines. Although 10 µg of protein was added into each well, the volume of lysate exceeded the well capacity for jetOPTIMUS and EndoFectin transfected samples with all three cell lines. With 1HAE, GFP expression with jetOPTIMUS was 39% greater than L3000 (*p* = 0.009). With 16HBE, only GFP expression with jetOPTIMUS was significantly different from the other samples. With NCI-H292 significantly greater signal was observed with L3000 compared to jetOPTIMUS and EndoFectin with no appreciable signal observed with FuGENE, EndoFectin, and CaP.

Transfected cell cultures were also dissociated and analyzed through flow cytometry at 48-hour post-transfection (Fig. 2C). For 1HAE cultures, no difference in transfection efficiencies of L3000, EndoFectin, and jetOPTIMUS transfections was observed. FuGENE, ViaFect, and CaP had the lowest transfection efficiencies. This was also observed in 16HBE and NCI-H292. However, in 16HBE, jetOPTIMUS yielded an 82% greater transfection efficiency compared to L3000. Descriptive statistics of transfection efficiency with each reagent are provided in Supplementary Table 1.

To characterize the cellular viability following transfection, Alamar Blue viability assays were performed at 24- and 48-hour post-transfection (Fig. 2D). Alamar Blue assays rely upon a redox reaction that converts the cell permeable dye, resazurin, to resorufin in metabolically active cells. This production of resorufin changes the fluorescence characteristics in proportion to the number of viable cells [24].

In 1HAE, cellular viability at 24- and 48-hour post-transfection was reduced with jetOPTIMUS and EndoFectin, consistent with our earlier findings of higher cell death with these reagents under light microscopy at 48-hour post-transfection. No significant change in cellular viability was observed with other reagents used. Similar to 1HAE, jetOPTIMUS and EndoFectin transfection resulted in significantly reduced viability compared to control in 16HBE with no significant change with any other reagents observed. However, at 48-hour post-transfection, L3000, ViaFect, jetOPTIMUS, and EndoFectin all demonstrated reduced cellular viability compared to control. In NCI-H292, L3000, ViaFect, jetOPTIMUS, and EndoFectin transfection resulted in significantly reduced viability compared to control at 24- and 48-hour post-transfection.

Moreover, cellular viability significantly increases between the 24 and 48-hour timepoints in the control samples of 1HAE, 16HBE, and NCI-H292 (*p* < 0.0001 for all three cell lines). This increase in cellular viability between time points was not observed with only jetOPTIMUS and EndoFectin transfection in all three cell lines. No increase was observed with L3000 in 16HBE, NCI-H292, and ViaFect in NCI-H292.

To improve transfection efficiency while preserving cellular viability, we tested four optimization techniques. Since viability with L3000 transfection was comparable to control in experiments above, we first assessed the doubling of the transfection complex in each culture well to assess the impact on transfection efficiency and cell viability. Second, given that viability often decreases after transfection, and certain protocols recommend changing media within 6–24 h [25, 26], we evaluated whether changing the media at 6 h would benefit transfection outcomes. Third, we tested a brief trypsin-EDTA pre-treatment, a technique previously shown to enhance transfection in polarized cultures [27], to determine if it would similarly benefit monolayer cell culture. Finally, since serum is known to interfere with DNA complex formation and reduce transfection efficiency [28, 29], we tested transfection in a reduced-serum medium (Opti-MEM) for 24 h.

**Fig. 3:**
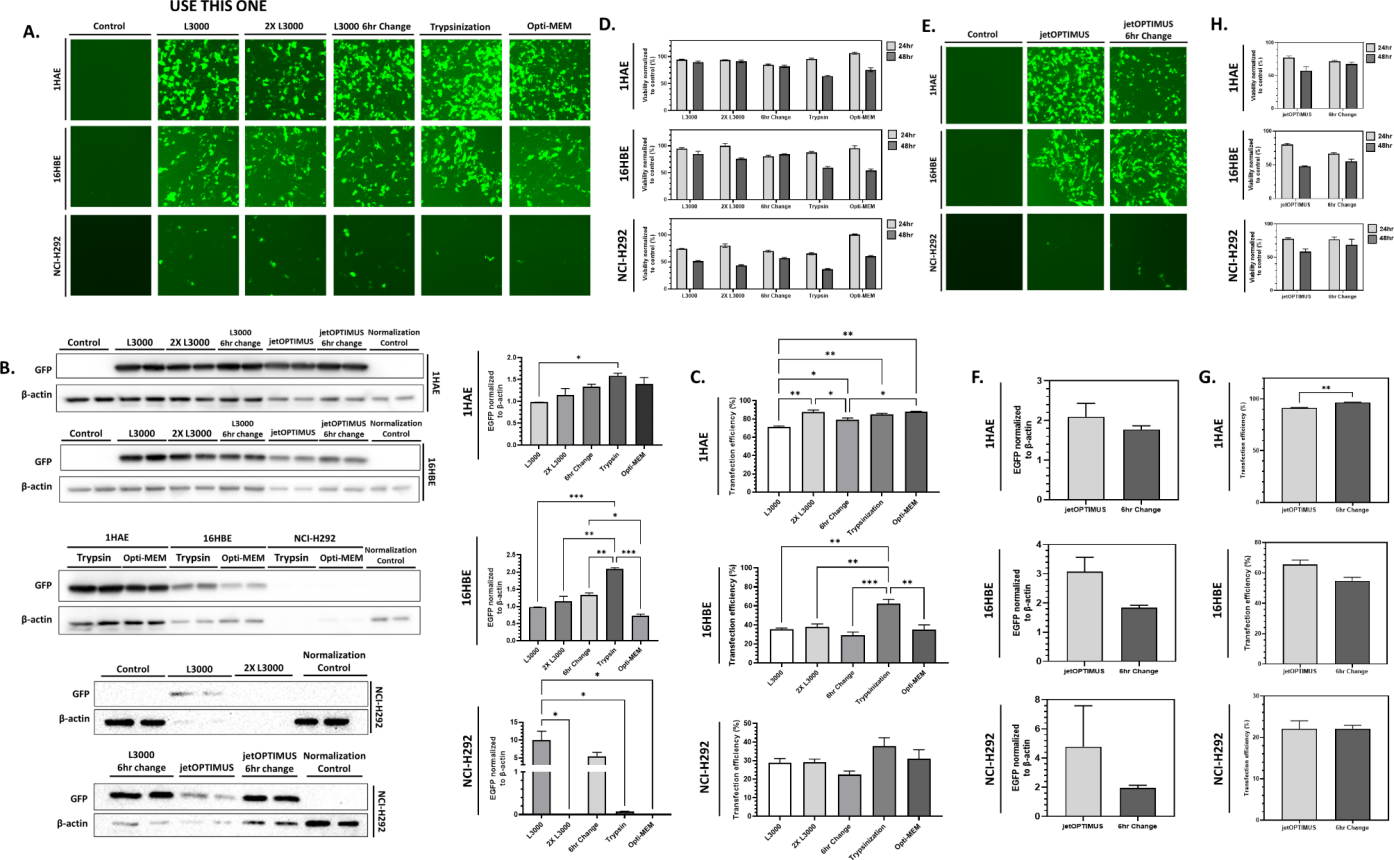
Transgene expression and cellular viability vary with different optimization techniques tested. (**A**) GFP microscopy of 1HAE, 16HBE, and NCI-H292 cell lines at 48-hour post-transfection. L3000 was used as the transfection reagent for all treatments. 2X L3000 represents treating the cells with double the transfection mixture. L3000 6 h change represents replacement of the transfection mixture containing media within 6 h post-transfection. Trypsinization represents two brief rinses of 0.25% trypsin-EDTA of the cell cultures prior to transfection. Opti-MEM represents incubating cells during transfection in Opti-MEM instead of complete DMEM. Images are at 100X magnification and representative of transfections done in duplicate, with the experiment conducted twice. (**B**) Western blot and analysis of 1HAE, 16HBE, and NCI-H292 cultures 48-hour post-transfection against GFP (26 kDa) and β-actin (42 kDa) as a loading control. Only comparisons with *p* > 0.05 are shown. (**C**) Flow cytometry analysis for transfection efficiency represented as the percentage of cells gated positive for GFP, compared to control. Data represents two independent transfection experiments. Only comparisons with *p* > 0.05 are shown. (**D**) Alamar Blue cell viability assay at 24- and 48-hour post-transfection, normalized to either 24- and 48-hour control. (**E**) GFP microscopy of 1HAE, 16HBE, and NCI-H292 cell lines at 48-hour post-transfection with jetOPTIMUS. 6 h change treatment represents replacement of the transfection mixture containing media within 6 h post-transfection. (**F**) Western blot and analysis with GFP expression normalized to β-actin (**G**) Flow cytometry analysis for transfection efficiency with jetOPTIMUS. Data represents two independent transfection experiments. Only comparisons with *p* > 0.05 are shown. (**H**) Alamar Blue cell viability assay at 24- and 48-hour post-transfection with the jetOPTIMUS normalized to either 24- or 48-hour control. For all experiments, * = *p* < 0.05, ** = *p <* 0.01, *** = *p* < 0.001. All experiments included two technical replicates and were repeated twice

Following these optimization techniques, differences in GFP expression were examined qualitatively through fluorescence microscopy (Fig. 3A). There appeared to be a slight reduction in signal when the transfection mixture was doubled (2X L3000 treatment) and an increase with trypsin pre-treatment in 1HAE. No obvious difference was observed between L3000, 6-hour media change, or incubation with Opti-MEM. With 16HBE, there was an apparent increase in GFP signal with trypsin pre-treatment and no obvious difference between the treatments. There were no clear differences across the treatments with NCI-H292. However, only L3000 and the 6-hour media change condition yielded transfections where signal could be observed through microscopy, though it was not clearly different from each other.

With western blotting against GFP of resolved whole cell-lysates of transfected 1HAE cells, there was no significant difference between the treatments tested (Fig. 3B). Variations in β-actin are due to the samples exceeding the well volume. With 16HBE, higher normalized GFP-expression was observed in trypsin pre-treatment, which was significantly greater than L3000, 2X L3000, 6-hour media change, and Opti-MEM media incubation. With NCI-H292, only L3000 had significantly greater normalized GFP expression compared to control.

Transfection efficiency, assessed by flow cytometry varied with optimization techniques (Fig. 3C). For 1HAE, all optimization techniques had greater transfection efficiency compared to L3000. With 16HBE, only trypsin pre-treatment had a significantly greater transfection efficiency compared to L3000. Additionally, trypsin pre-treatment resulted in a greater transfection efficiency compared to 2X L3000, 6-hour media change, and Opti-MEM media incubation. NCI-H292 exhibited no significant differences in transfection efficiency across treatments. Descriptive statistics of transfection efficiency with each optimization technique are provided in Supp. Table 2.

In Fig. 3D, at 24-hour post-transfection in 1HAE, 6-hour media change had the lowest cellular viability, but trypsin pre-treatment and Opti-MEM incubation had the lowest viability at 48-hour post-transfection. Similarly, at 24-hour post-transfection in 16HBE, 6-hour media change had the lowest cellular viability. Trypsin pre-treatment and Opti-MEM incubation was the lowest across all treatments at 48-hour post-transfection. In NCI-H292, all treatments, except for Opti-MEM incubation, reduced cellular viability at 24-hour post-transfection. 48-hour post-transfection, all treatments had significantly lower viability compared to control. Only trypsin pre-treatment resulted in an obvious decrease in culture confluency in 1HAE and 16HBE; no differences between treatments was observed with NCI-H292 (Supp. Figure 2A).

Transfection with jetOPTIMUS showed similar or greater efficiency compared to L3000 (Fig. 1); however, it also led to significant cell death and reduced viability, dependent on the cell line used. To address this, we investigated whether changing the media 6 h post-transfection could improve cell viability while maintaining transfection efficiency with this reagent. This approach is based on the observation that some transfection reagents can become cytotoxic if left in prolonged contact with cells [30].

With GFP fluorescence microscopy of transfection with jetOPTIMUS, an observable reduction in GFP signal was observed with 6-hour media change in 1HAE (Fig. 3E). However, no distinct observable differences in signal were observed in 16HBE or NCI-H292. GFP expression assessed via western blotting was not significantly different between jetOPTIMUS and 6-hour media change in all cell lines (Fig. 3F). 6-hour media change had significantly higher transfection efficiency in 1HAE, as analyzed via flow cytometry, though there was no difference in 16HBE or NCI-H292 (Fig. 3G). Descriptive statistics of transfection efficiency with jetOPTIMUS and media change are provided in Supplementary Table 3.

In 1HAE, jetOPTIMUS transfection reduced cellular viability compared to control, while 6-hour media change also led to reduced viability compared to jetOPTIMUS. At 48-hour post-transfection, both jetOPTIMUS and the 6-hour media change showed significantly lower viability than control, with no difference between the two treatments. In 16HBE, similar trends were observed, with both jetOPTIMUS and the 6-hour media change significantly reducing viability compared to control at both 24- and 48-hour post-transfection. However, the 6-hour media change resulted in greater viability compared to jetOPTIMUS at 48 h. In NCI-H292, there were no viability differences between jetOPTIMUS and media change at both 24- and 48-hour post-transfection, but both treatments were lower than control at both time points. No obvious difference in culture confluency was observed across the three cell lines with jetOPTIMUS and 6-hour media change, but both treatments resulted in significantly lower confluency compared to control (Supp. Figure 2B).

## Discussion

Transfections are a commonly used method for gene delivery in airway epithelial research. Gene delivery methods have been investigated and optimized in therapy, such as the delivery of correct copies of the cystic fibrosis transmembrane conductance regulator (*CFTR*) [31]. Focusing on basic biomedical research, chemical transfection techniques represent generally cost-effective and easy-to-implement method to study the function of genes and gene products [9]. In this study, we examined the transfection efficiency of an EGFP-expressing plasmid into three commonly used airway epithelial cell lines with six different chemical transfection reagents.

We observed that transfection efficiency was significantly different across 1HAE, 16HBE, and NCI-H292 cell lines. These differences may be due to the differential expression of various deoxyribonucleases (DNases) across cell lines. Hoffman et al. observed that inhibition of DNAse I localized to the cytosol and nucleus increased transfection efficiency two-fold [32]. Howell et al. observed that the increased expression of DNase II, present in the lysosome, correlated with decrease in transfection efficiency [33]. In our hands, using calcium phosphate as a transfection reagent yielded limited success. Interestingly, our results showed an inverse relationship between transfection efficiency and DNase I expression across cell lines: 1HAE had both the highest transfection efficiency and the highest DNase I expression. DNase II expression was comparable in 1HAE and NCI-H292 but was lower in 16HBE. These observations suggest the presence of other cell-specific barriers to gene delivery across the tested cell lines, such as cellular internalization, intracellular trafficking, and endosomal escape [34]. These specific characteristics were assessed by Figueroa et al. who observed that easily transfectable SK-BR3 cells took up DNA more rapidly and showed greater expression after chloroquine treatment due to enhanced endosomal escape. In contrast, hard-to-transfect CT26 cells retained most of the transfected DNA in acidic organelles, with minimal nuclear localization. They suggest that CT26’s low transfection efficiency may be due to abundant cell surface mucins which confer a physical barrier against transfection, high exocrine activity, and increased sensitivity to foreign materials [35]. Furthermore, complexes internalized via clathrin-mediated endocytosis are trafficked to the endolysosomal pathway, where endosomal escape can occur. This pathway is observed in 293T cells, which show higher transfection efficiency compared to CHO cells. In CHO cells, complexes are internalized through caveolin-mediated endocytosis, rendering them transfection-incompetent [36]. Differences in cellular proliferation rates can also impact transfection efficiency. Studies suggest that transfection using cationic lipids or polymers yields higher levels of reporter gene expression in actively dividing cells [37, 38]. A significant barrier may be the entry of plasmid into the nucleus; mitosis facilitates this process by allowing intact plasmid access to the intranuclear compartment necessary for transgene expression [39]. 16HBE cells have a doubling time of approximately 50 h at lower passages [40], while NCI-H292 cells double in approximately 30 h [41]. The doubling time for 1HAE has not been reported. Although transfection efficiencies with L3000 were comparable between 16HBE and NCI-H292, these observations suggest that other factors may play a larger role in influencing transfection outcomes. Considering our observations and previous studies, it is important for researchers to optimize transfection in their cell line of interest rather than solely relying on previous successes in similar cell lines.

We also observed that the transfection efficiency and cell viability following transfection were dependent on the transfection reagent used. In all three cell lines tested, L3000 and jetOPTIMUS yielded the greatest transfection efficiency, with L3000 having a lower impact to cellular viability 24- and 48-hour post-transfection in the three cell lines tested. However, L3000 was still not benign; transfections with it reduced cellular viability compared to control at 48-hour post-transfection in all cell lines tested. Cationic lipids, which include L3000, have been observed to demonstrate toxicity through the induction of apoptosis by causing mitochondrial and oxidative stress and DNA damage [42]. Other reagents such as cationic polymer based reagents such as PEI have shown to induce non-specific gene expression changes that could result in apoptosis of target cells, reasoning for the lower viability [43, 44]. Furthermore, complexes with highly positive zeta potentials and smaller complexes are associated with increased cytotoxicity [45–47]. For example, Lipofectamine 2000 was reported to generate lipoplexes with diameters of 147–380 nm and zeta potentials between + 5 to + 13 mV whereas non-liposomal complexes, such as PEI, can range from 81 to over 2000 nm with zeta potentials from + 25 to + 40 mV [45, 48]. With L3000 complexed with plasmid, mean hydrated diameter of the complexes were in the range of 400–500 nm and zeta potential closer to neutral [49]. However, due to the proprietary nature of the reagents used in this study, we can only speculate on the characteristics of the resulting complexes. Further investigation into the relationship between complex size, zeta potential, and their impact on airway epithelial cell viability is warranted.

Internalized complexes of cationic lipids or polymers with DNA fuse with the endocytic compartment, and without endosomal escape, they are trafficked to late endosomes for degradation [34]. Therefore, endosomal escape is essential for gene delivery. One mechanism through which this may occur is the “proton sponge” theory, where protonation of amino groups in cationic polymers such as PEI, buffers the acidic endosomal environment, resulting in proton and chloride influx and subsequent osmotic swelling and disruption of the endosome [50]. Other mechanisms include cationic lipids and polymers interacting with lipids of the endosomal membrane leading to membrane fusion or pore formation, allowing for escape [51]. Frequency of endosomal escape can vary based on transfection reagent used. For instance, cationic lipids such as vectamidine and DMRIE-C has been observed to disrupt endosomes more than lipofectin, which correlated to their higher transfection efficiencies [52]. Similarly, polymer-based reagents like JetPEI demonstrate higher endosomal escape frequencies compared to lipid-based reagents like lipofectin, attributed to their pH-dependent pore-forming activities [53]. Polyplexes, formed between a cationic polymer and siRNA, and liposomes, formed between Lipofectamine 2000 and siRNA, were also observed to have differential endosomal escape rates, but both relying on proton sponge mechanism for escape [54]. These observations suggest that efficiency of gene delivery can also depend significantly on the transfection reagent’s ability to induce endosomal escape.

To mitigate the cellular toxicity of L3000 and jetOPTIMUS, we conducted experiments where we changed the media six hours post-transfection. This modification may minimize the exposure time of excess transfection reagents to the cell culture, resulting in reduced apoptosis and increased transfection efficiency. However, the effects of this on cellular viability were generally not significant, with exceptions of increased viability in 16HBE with jetOPTIMUS and NCI-H292 with using L3000 at 48-hour post-transfection. In respect to transfection efficiency, only in 1HAE was an increase in transfection efficiency observed, while no significant difference was observed in 16HBE or NCI-H292. The toxicity of these reagents is likely independent of exposure time and consequently, changing the media following transfection is not beneficial.

Two brief 0.25% trypsin-EDTA rinses significantly improved transfection efficiency with L3000 in both 1HAE and 16HBE. This is consistent with findings reported by Rybakovsky et al. They observed improved transfection efficiency and VP40 protein expression with a pre-treatment of trypsin-EDTA, which was hypothesized to allow for greater reception for fusion with the membrane and disruption of paracellular tight junctional barriers to increase surface area for exposure via the proteolysis of integral proteins [27]. However, in this study, submerged, undifferentiated, airway epithelial monolayers were used that have limited culture thickness. Although these cells express junctional proteins, their cleavage and subsequent increase in surface area may be limited due to the thinness of these cultures. We hypothesize that trypsin-EDTA pre-treatment can increase transfection efficiency by cleaving cell surface glycosaminoglycans (GAGs). Bronchial and tracheal cell lines express GAGs, such as heparan sulfate and chondroitin sulfate, on their cell surface [55, 56]. These molecules contribute to barrier function and modulation of cell signalling within the airway epithelium [57–59]. As polyanionic molecules, GAGs can bind to DNA-reagent complexes, potentially sequestering them and preventing efficient cellular uptake [60], and therefore reducing transfection efficiency. Transgene expression was increased by 3- to 25-fold in heparan sulfate and chondroitin sulfate-deficient CHO cells compared to wild-type cells, depending on the cationic polymer or lipid used [60]. Furthermore, transfection using dendrimer-pDNA complexes showed a tenfold increase in plasmid DNA nuclear uptake and a 2.6-fold improvement in transfection efficiency when GAGs were absent from the cell surface [61]. Trypsin-EDTA incubation has been demonstrated to release significant amounts of glycopeptides into the culture medium, with heparan sulfate and chondroitin sulfate predominating in the released material [62]. Trypsin-EDTA pre-treatment therefore can contribute to the reduction of surface-bound GAGs, which can promote greater interaction between the cell surface and DNA-reagent complexes for uptake.

Transfecting with Opti-MEM instead of serum-supplemented DMEM did not yield any promising effect on improving transfection efficiency nor cell viability. Transfection with trypsin-EDTA pre-treatment could be a potential technique that can be used to enhance transfection efficiency, especially in cultures that are difficult to transfect.

Future investigations should be focused on examining the efficacy of these reagents on differentiated cultures of these airway epithelial cell lines. These cultures are desirable as they more closely resemble the airway epithelial structure and function in vitro [63]. However, the presence of mucus and cilia, tight junctions, and increased quiescence of these differentiated tissues may pose additional challenges in chemical transfection.

## Conclusions

With investigations of the airway epithelium involving the use of chemical transfections, consideration should be given to the cell line or cellular source, transfection reagents used, and brief trypsin-pre-treatment to increase transfection efficiency. The optimization of chemical transfection is often tedious and time-consuming. Here, we have provided the results of our optimization efforts, including various assays to characterize transgene expression and cellular viability in three commonly used airway epithelial cell lines, employing six commonly used or commercially available transfection reagents. We observed that 1HAE generally had higher transfection efficiencies followed by 16HBE and NCI-H292. L3000 yielded the greatest transfection efficiency with the least impact on cellular viability, making it preferable for the cell lines tested. However, jetOPTIMUS had higher or comparable transfection efficiency in 1HAE and 16HBE, at the expense of reduced cellular viability, requiring additional optimization. Doubling the transfection mixture or changing the media at 6-hour post-transfection was generally not beneficial. However, a brief pre-treatment with trypsin-EDTA improved transfection efficiency in 1HAE and 16HBE. We hope these results can guide researchers in navigating through the intricacies of transfection procedures.

## Electronic supplementary material

Below is the link to the electronic supplementary material.

Supplementary Material 1

Supplementary Material 2

## Data Availability

Raw and analyzed data obtained for this current study are available from the corresponding author upon reasonable request.

## Abbreviations

ANOVA: Analysis of variance
CaP: Calcium phosphate transfection
DMEM: Dulbecco’s modified Eagle’s medium
EDTA: Ethylenediaminetetraacetic acid
EGFP: Enhanced green fluorescent protein
L3000: Lipofectamine 3000
SV40: Simian virus 40
