# Supplementary material for: Optimization of chemical transfection in airway epithelial cell lines
Source: BMC Biotechnol. 2025 Jan 23;25:10. doi: 10.1186/s12896-025-00945-x (PMC11761256; doi:10.1186/s12896-025-00945-x)

Uncropped blots

Guo TJF et al. Optimization of transfection in airway epithelial cell lines

Figure 1B

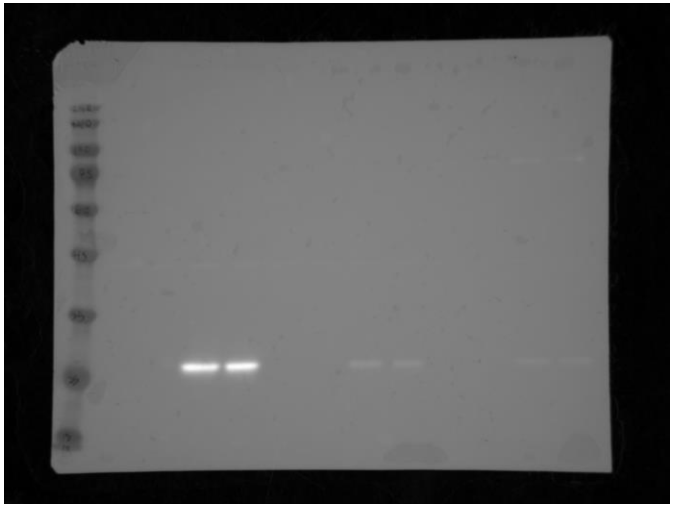

EGFP

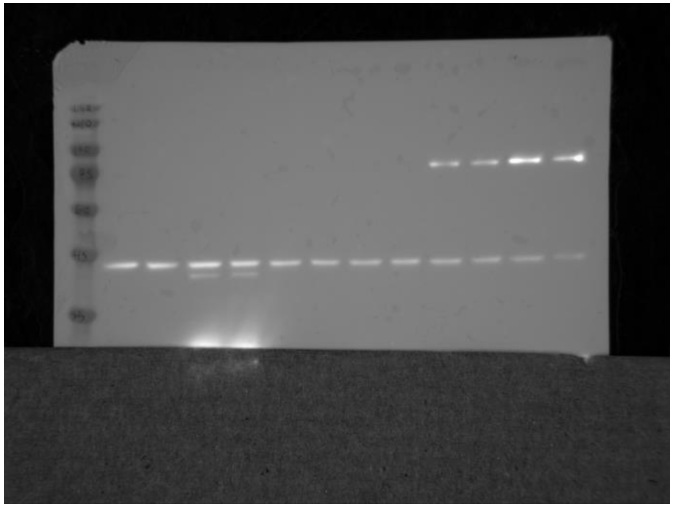

β-actin

Figure 1E

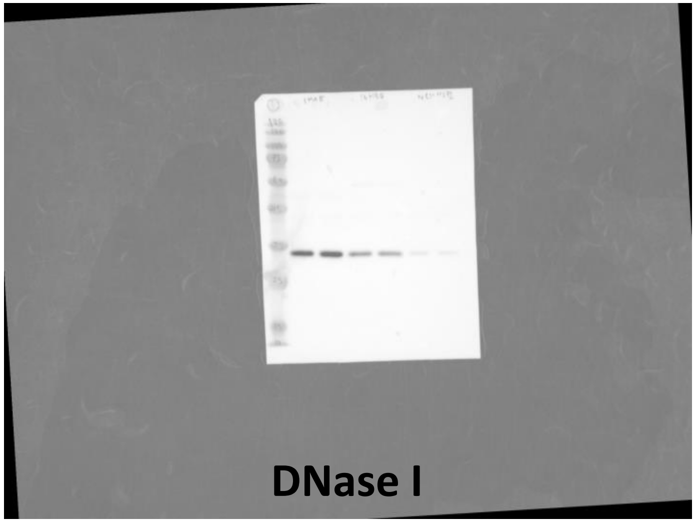
DNase I

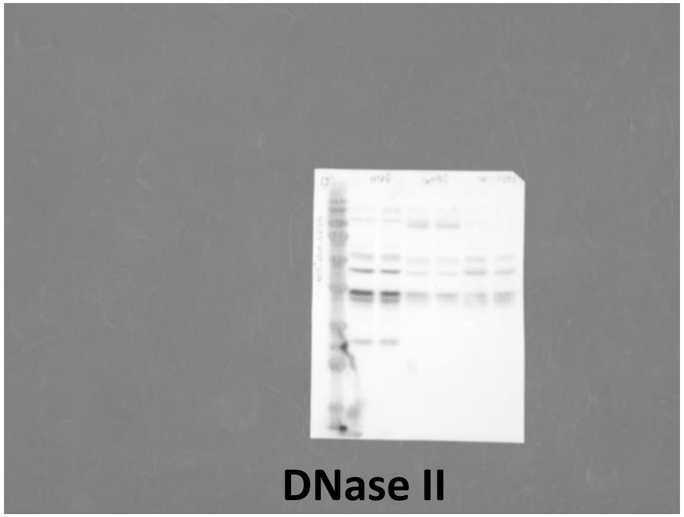
DNase II

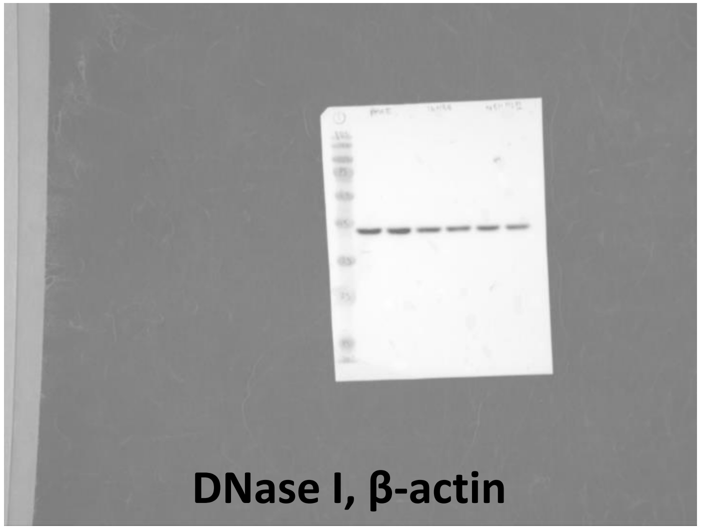
DNase I, β-actin

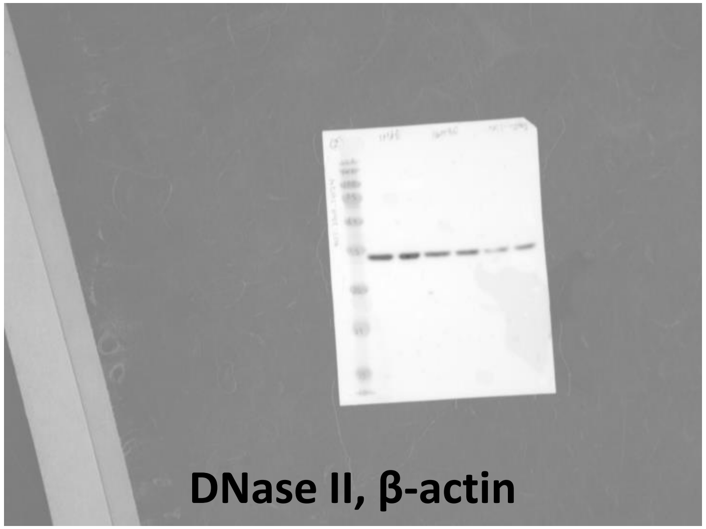
DNase II, β-actin

Figure 2B

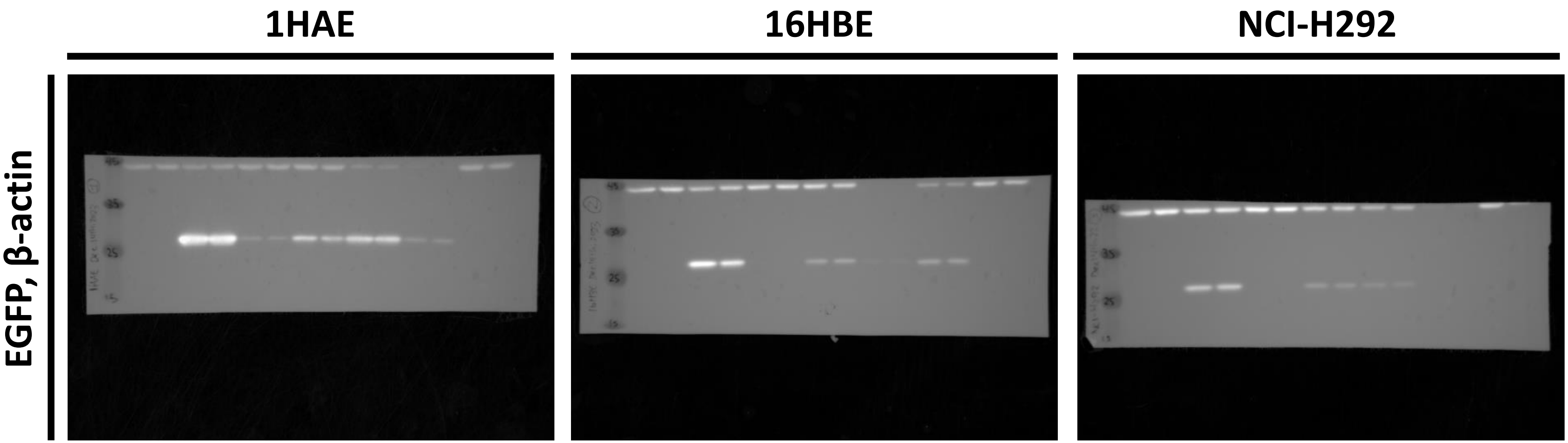

Figure 3B

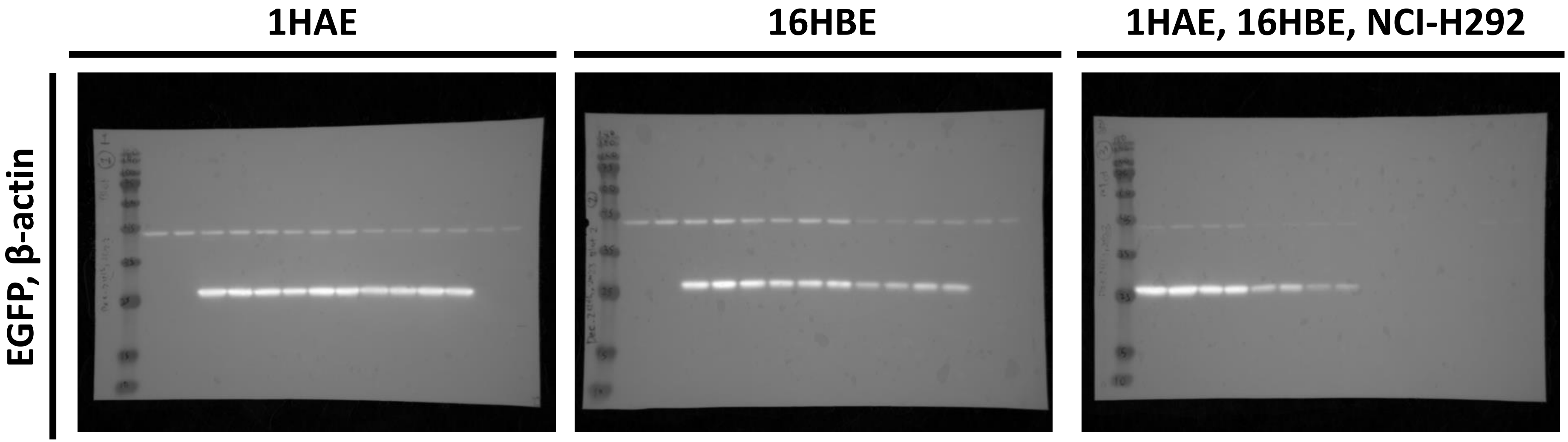

Figure 3B, continued

NCI-H292, blot 1 and 2, EGFP and Actin

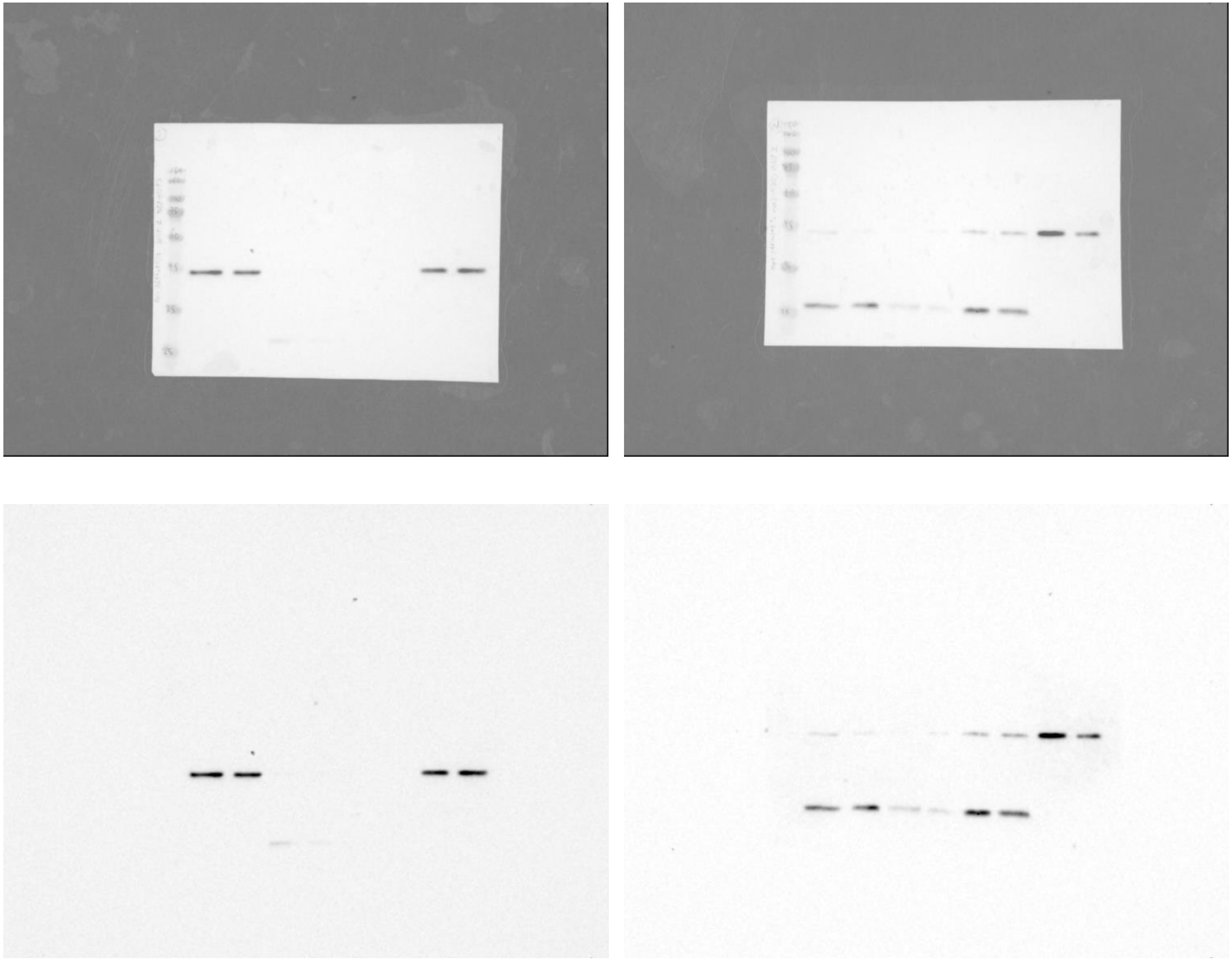

Supplement: Supplementary file 1 — Supplementary Material 1 [file 12896_2025_945_MOESM1_ESM.pdf]
