# Supplementary material for: Optimization of chemical transfection in airway epithelial cell lines
Source: BMC Biotechnol. 2025 Jan 23;25:10. doi: 10.1186/s12896-025-00945-x (PMC11761256; doi:10.1186/s12896-025-00945-x)
Supplement: Supplementary file 2 — Supplementary Material 2 [file 12896_2025_945_MOESM2_ESM.docx]

**
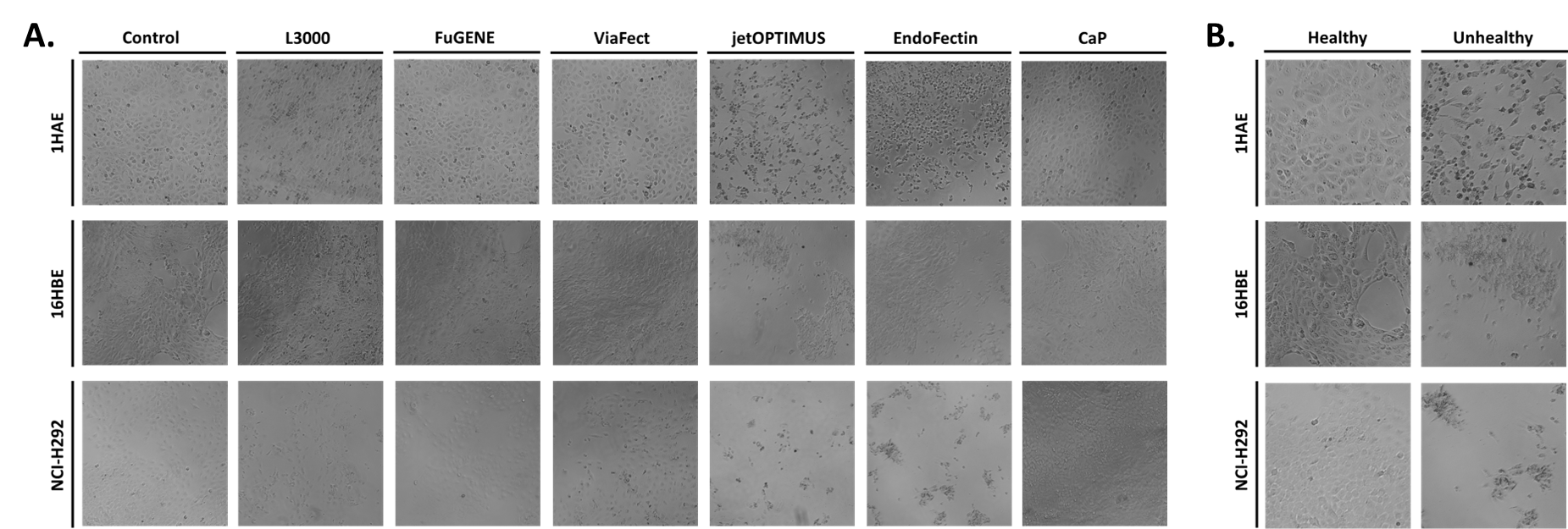
**

**Supp. Fig. 1. A.** Transmitted light microscopy images of 1HAE, 16HBE, and NCI-H292 cultures 48-hour post-transfection with various transfection reagents. Each image represents transfections done in duplicate over two independent experiments and taken at 100X magnification. **B.** Light microscopy images of 1HAE, 16HBE, and NCI-H292 cultures considered to be healthy and unhealthy.

| **Supp. Table 1 Flow cytometry transfection efficiency with various reagents** | | | | | | |
| --- | --- | --- | --- | --- | --- | --- |
| **Transfection Efficiency, Mean ± SEM** | | | | | | |
|  | **L3000** | **FuGENE** | **ViaFect** | **jetOPTIMUS** | **EndoFectin** | **CaP** |
| **1HAE** | 76.11 ± 3.22 | 36.33 ± 0.10 | 22.71 ± 0.58 | 90.68 ± 4.197 | 62.01 ± 7.86 | 1.95 ± 0.19 |
| **16HBE** | 35.48 ± 1.21 | 7.98 ± 0.19 | 22.03 ± 1.38 | 64.58 ± 3.17 | 31.76 ± 1.73 | 2.17 ± 0.08 |
| **NCI-H292** | 28.87 ± 2.23 | 4.59 ± 0.32 | 6.93 ± 0.46 | 22.55 ± 1.16 | 17.49 ± 0.97 | 1.24 ± 0.18 |

**Supp. Table 1.** Transfection efficiency as assessed via flow cytometry of 1HAE, 16HBE, and NCI-H292 cultures transfected using L3000, FuGENE, ViaFect, jetOPTIMUS, EndoFectin, and CaP. Transfection efficiency is represented as the percentage of cells gated for positive for GFP signal, compared to control.


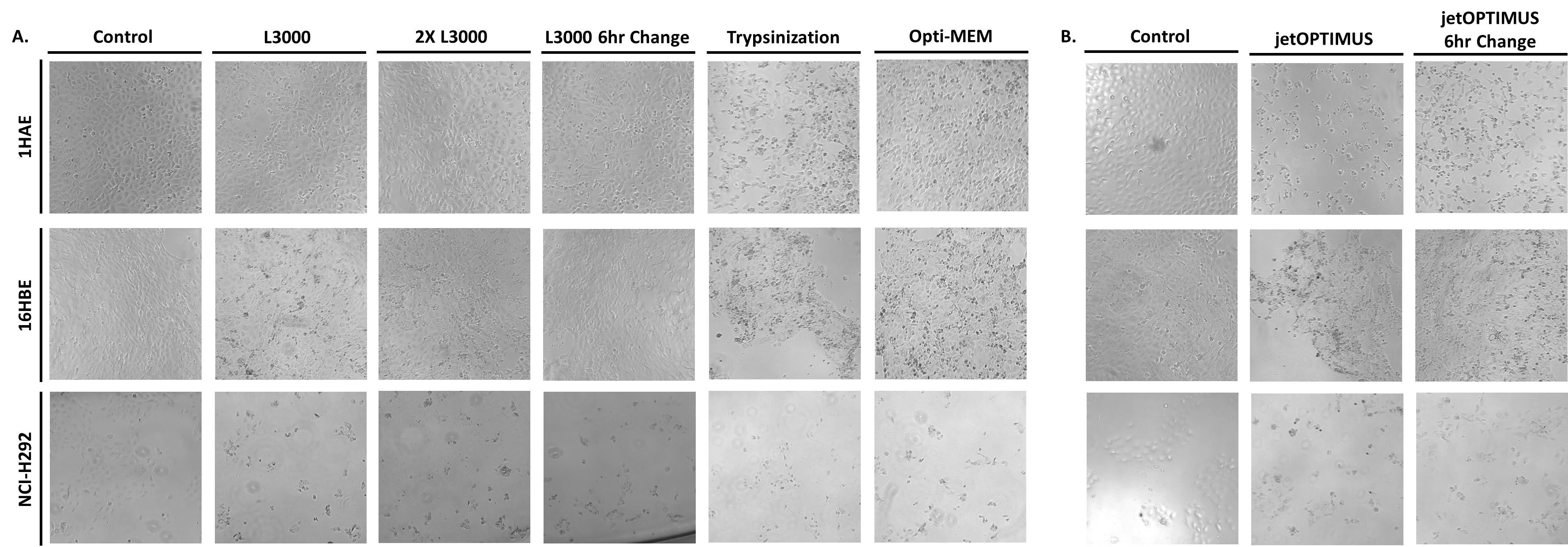


**Supp. Fig. 2.** Transmitted light microscopy images of 1HAE, 16HBE, and NCI-H292 cultures 48-hour post-transfection following various optimization techniques. **A.** L3000 was used as the transfection reagent for these treatments. The following treatments were performed: 2X L3000 - double the transfection mixture. L3000 6 hr change – media change 6 hours post-transfection. Trypsinization - two brief rinses of 0.25% trypsin-EDTA of the cell cultures prior to transfection. Opti-MEM – incubation of cells during transfection in Opti-MEM instead of complete DMEM. Images are at 100X magnification and representative of transfections done in duplicate, with the experiment conducted twice. **B.** 1HAE, 16HBE, and NCI-H292 cultures subjected to jetOPTIMUS transfection, and 6-hour media change post-transfection. Each image represents transfections done in duplicate over two independent experiments and taken at 100X magnification.

| **Supp. Table 2 Flow cytometry transfection efficiency following various optimization techniques** | | | | | |
| --- | --- | --- | --- | --- | --- |
| **Transfection Efficiency, Mean ± SEM** | | | | | |
|  | **L3000** | **2X L3000** | **L3000 6 hr change** | **Trypsinization** | **Opti-MEM** |
| **1HAE** | 72.31 ± 0.98 | 87.79 ± 2.01 | 79.30 ± 1.78 | 85.15 ± 1.05 | 88.25 ± 0.21 |
| **16HBE** | 35.48 ± 1.21 | 37.76 ± 3.18 | 29.38 ± 3.13 | 62.55 ± 4.31 | 34.82 ± 5.19 |
| **NCI-H292** | 28.87 ± 2.23 | 29.09 ± 1.66 | 22.44 ± 1.90 | 37.79 ± 4.52 | 30.99 ± 4.86 |

**Supp. Table 2.** Transfection efficiency assessed via flow cytometry with various optimization techniques.

| **Supp. Table 3 Transfection efficiency with jetOPTIMUS and 6 hr media change** | | |
| --- | --- | --- |
| **Transfection Efficiency, Mean ± SEM** | | |
|  | **jetOPTIMUS** | **jetOPTIMUS, 6hr change** |
| **1HAE** | 91.51 ± 0.29 | 96.74 ± 0.36 |
| **16HBE** | 65.62 ± 2.87 | 54.79 ± 2.37 |
| **NCI-H292** | 22.08 ± 1.93 | 22.11 ± 0.79 |

**Supp. Table 3.** Transfection efficiency following transfection using jetOPTIMUS and changing media 6-hr post-transfection.
